# Pharmaceutical compounding and storage of faricimab in a syringe for intravitreal injection do not impair stability and bi-specific binding properties

**DOI:** 10.1186/s40942-023-00507-3

**Published:** 2023-11-07

**Authors:** Øystein Kalsnes Jørstad, Stian Foss, Torleif Tollefsrud Gjølberg, Simone Mester, Mari Nyquist-Andersen, Magne Sand Sivertsen, Dag Fossum, Espen Gleditsch, Morten Carstens Moe, Jan Terje Andersen

**Affiliations:** 1 Department of Ophthalmology, Oslo University Hospital, Oslo, Norway; 2 Department of Pharmacology, Oslo University Hospital and University of Oslo, Oslo, Norway; 3 Department of Immunology, Oslo University Hospital and University of Oslo, Oslo, Norway; 4 Precision Immunotherapy Alliance (PRIMA), University of Oslo, Oslo, Norway; 5 The Hospital Pharmacy Oslo, Ullevål, Norway; 6 Institute of Clinical Medicine, Faculty of Medicine, University of Oslo, Oslo, Norway

**Keywords:** Intravitreal injection, Faricimab, Compounding pharmacy, Prefilled syringes, Bi-specific binding

## Abstract

**Background:**

Intravitreal injection (IVI) of antibody biologics is a key treatment approach in ophthalmology. Pharmaceutical compounding and storage of prefilled syringes for IVI must take place without impairing the structure and function of the biologics. This study investigated the effect of withdrawing and storing the therapeutic antibody faricimab (Vabysmo, Roche, Basel, Switzerland) in the Zero Residual silicone oil-free, 0.2-mL syringe (SJJ Solutions, The Hague, the Netherlands).

**Methods:**

To assess the effect of syringe withdrawal on faricimab, we compared samples from syringes prepared at day 0 with samples taken directly from faricimab vials. To assess the effect of syringe storage on faricimab, we kept prefilled syringes in the dark at 4 ^o^C for 7, 14, or 37 days and compared samples from these syringes with day 0. We measured protein concentration (with spectrophotometry), stability and integrity (with sodium dodecyl sulfate-polyacrylamide gel electrophoresis (SDS-PAGE), size-exclusion chromatography (SEC), and melting temperature (Tm)), as well as binding of faricimab to its cognate antigens: vascular endothelial growth factor A (VEGF-A) and angiopoietin-2 (Ang-2) (with enzyme-linked immunosorbent assay (ELISA)).

**Results:**

Faricimab migrated in line with its expected molecular mass under both reducing and non-reducing conditions for all time points when analyzed with SDS-PAGE, without any sign of degradation products or aggregation. The SEC elution profiles were identical for all time points. There were slight variations in Tm for different time points compared to day 0 but without consistent relationship with storage time. ELISA did not detect differences in VEGF-A or Ang-2 binding between time points, and faricimab did not bind the neonatal Fc receptor.

**Conclusions:**

Withdrawal and storage of faricimab in syringes for up to day 37 did not impair the structure and bi-specific binding properties of the therapeutic antibody.

**Supplementary Information:**

The online version contains supplementary material available at 10.1186/s40942-023-00507-3.

## Introduction

Intravitreal injection (IVI) of biologics that inhibit vascular endothelial growth factor A (VEGF-A) is a key treatment approach for retinal diseases in contemporary ophthalmology. IVI involves withdrawing the drug from a vial into a syringe, a task preferably entrusted to a compounding pharmacy to optimize hygiene standards, save clinician time, and allow for secure splitting of vials into multiple syringes. However, antibody biologics are delicate proteins, and it is imperative that pharmaceutical compounding and storage of prefilled syringes take place without impairing the drug properties. Additionally, the syringe itself must not only preserve the drug but also accurately deliver it intravitreally, typically as a 50-µL dose. In the latter regard, there are significant differences between commonly used syringes [1].

We have previously shown that the prevailing antibody biologics in ophthalmology, namely bevacizumab (Avastin, Roche, Basel, Switzerland), ranibizumab (Lucentis, Novartis, Basel, Switzerland), and aflibercept (Eylea, Bayer, Leverkusen, Germany), can be stored for up to 30 days without compromising their functional binding or transport properties [2]. A new therapeutic antibody for IVI is now available: faricimab (Vabysmo, Roche). It differs from the former antibody biologics by not only inhibiting VEGF-A but also angiopoietin-2 (Ang-2) [3]. This bi-specific feature may contribute to its favorable efficacy and durability in neovascular age-related macular degeneration and diabetic macular oedema [4, 5].

Amid pharmaceutical compounding of prefilled syringes for IVI, the effect of this practice on faricimab and its unique mechanism of action remains unknown. This study investigated whether faricimab can be safely withdrawn and stored in syringes for up to 37 days.

## Methods

**Fig. 1:**
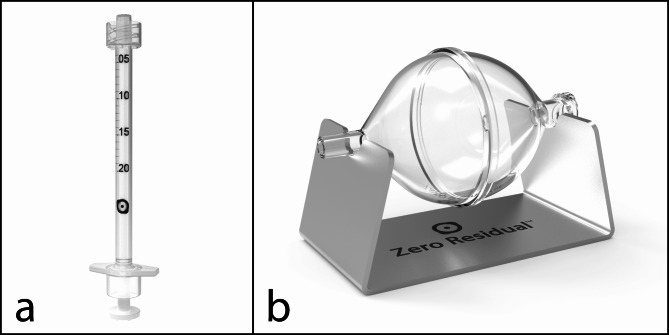
The Zero Residual silicone oil-free, 0.2-mL syringe **(a)** and Zero Residual Bubble Adapter **(b)**. Note, the images are not to scale

We prefilled Zero Residual silicone oil-free, 0.2-mL syringes (SJJ Solutions, The Hague, the Netherlands) under aseptic conditions at The Hospital Pharmacy Oslo, Ullevål in accordance with the ISO 14,644 guidelines and EU Good Manufacturing Practice. To facilitate withdrawal of faricimab from vials into syringes, we used Zero Residual silicone oil-free filter needles and the auxiliary Zero Residual Bubble Adapter (SJJ Solutions) (Fig. 1b), a silicone oil-free device designed for air-free filling of the Zero Residual syringe. We withdrew 3 faricimab vials at a time into the adapter and then split the content by withdrawal from the adapter into 10 syringes. We prepared a total of 72 prefilled syringes, which we packed separately in sterile plastic bags. We did not observe clogging, precipitation, leakage, or other problems after prefilling the syringes.

To assess the effect of syringe *withdrawal* on faricimab, we compared syringes (Fig. 1a) prepared at day 0 with a mounted needle (D0-n) to samples measured directly from faricimab vials. We prepared vial samples by pipetting the content of faricimab vials directly into LoBind Eppendorf tubes and aliquoting the resulting samples into 9 individual aliquots, which we subsequently treated in the same manner as the syringe samples. To assess the effect of syringe *storage* on faricimab, we kept prefilled syringes in the dark at 4 ^o^C for either 7, 14, or 37 days, and compared faricimab from these syringes with D0-n. Notably, one may choose to store syringes with mounted needles following compounding, but this prevents complete sealing and instead leads to slight exposure of faricimab to air during storage. To address whether such air exposure affects the stability of faricimab, we stored day-7 syringes with either 30-G, 13-mm Zero Residual needles (SJJ Solution) (D7-n) or Zero Residual Luer Lock caps (SJJ Solution) (D7-c). The latter completely sealed the syringes and prevented any air exposure. For syringes stored for 14 days (D14-c) or 37 days (D37-c), we always used Luer Lock caps.

### Concentration measurements

We used a DeNovix DS-11+ Spectrophotometer (DeNovix Inc., Wilmington, DE) to measure the faricimab concentration in each sample. We set the spectrophotometer to calculate the concentration on the basis of a molecular mass of 146.46 kDa and extinction coefficient of 248,965 M^− 1^ cm^− 1^, which we obtained from the Expasy Bioinformatics Resource Portal, assuming all cysteine residues form disulfide bonds, as the buffer in question is non-reducing. We performed the measurements in duplicates and averaged them before plotting. We washed and blanked the instrument with dH_2_O between time points. Note, 1:100 dilution designates dilution in sterile PBS. We used values from the concentration measurements for further analytical experiments.

### SDS-PAGE analyses

We ran the samples from vial, D0-n, D7-n and -c, D14-c, and D37-c under non-reducing and reducing conditions using sodium dodecyl-sulfate polyacrylamide gel electrophoresis (SDS-PAGE). The samples were prepared by diluting 2-µg protein in distilled water and BoltTM LDS loading buffer (Thermo Fisher Scientific, Waltham, MA), both with and without 10X BoltTM Sample Reducing Agent (Thermo Fisher Scientific). We heated the samples with the reducing agent for 5 min at 95 °C. We then applied the samples to 12% BoltTM Bis-Tris Plus gels (Thermo Fisher Scientific) before running them for 22 min at 200 V. We used a Spectra Multicolor High Range Protein Ladder (Thermo Fisher Scientific) for size comparison at the day of the study. We ran the gels again 5 days after the study day, next to the same protein ladder to confirm the migration of proteins. We visualized the proteins by Coomassie staining (Bio-Rad, Hercules, CA) and acquired the images using a Gel-Doc instrument (Bio-Rad Laboratories). The samples from the different time points were run in separate chambers.

### Size-exclusion chromatography (SEC)

We performed SEC using an ÄKTA Avant 25 instrument (GE Healthcare, Chicago, IL) coupled with a Superdex 200 Increase 10/300 GL column (GE Healthcare, Chicago, IL). We prepared 600-µg faricimab in a total volume of 100 µL, of which we injected 77 µL on the column using an autosampler (Spark Holland B.V., Emmen, the Netherlands). The sample compartment was kept at 4 ^o^C throughout the experiment. Samples were run in an alternate manner to avoid bias due to storage in the sample compartment.

### Nano differential scanning fluorimetry (DSF)

We used a Prometheus NT.48 instrument (NanoTemper Technologies GmbH, München, Germany) to measure the melting temperature by increasing the temperature by 1 °C/min from 20 to 95 °C. Undiluted samples were drawn into capillaries and prepared in duplicates. We determined the melting temperature (Tm), at which half of the protein unfolded, by deducing the first derivative in the PR.ThermControl (NanoTemper Technologies GmbH) software.

### Enzyme-linked immunosorbent assay (ELISA): Ang-2 and VEGF binding properties

We coated 96-well EIA/RIA 3590 plates (Corning Costar) with 100-µl 0.5-µg/mL human VEGF165 (Sino Biological, Beijing, China) or 100-µL 2.0-µg/mL His-tagged Ang-2 (Sino Biologics) followed by incubation overnight at 4 °C. The plates were blocked for 2 h at room temperature (RT) with 250-µl 4% skimmed milk powder (Sigma-Aldrich, Saint-Louis, MO) dissolved in PBS (Sigma-Aldrich) (S/PBS), followed by washing 4 times with PBS containing 0.05% Tween20 (T) (Sigma-Aldrich). Then 100-µl titrated amounts of faricimab was diluted in S/PBS/T, starting at 2.0 µg/mL for VEGF and 8.0 µg/mL for Ang-2, added to the plates in duplicates, and incubated at RT for 1 h on a shaker. Following washing, we detected bound faricimab by adding 100-µl alkaline phosphatase (ALP)-conjugated goat anti-human Fc antibody (Sigma-Aldrich), incubating for 1 h on a shaker, washing a final round, and subsequently adding 100-µl ALP substrate (1 mg/mL) dissolved in diethanolamine buffer. The absorbance at 405 nm was measured using a Sunrise spectrophotometer (Tecan, Männedorf, Switzerland).

### ELISA: FcRn binding properties

Antibodies link recognition of their cognate antigens via the fragment antigen binding (Fab) arms and binds to effector molecules via the constant Fc fragment. Most antibody biologics, including aflibercept and bevacizumab, contain a Fc fragment derived from the IgG1 subclass, which enables binding to FcRn. This binding occurs in a strictly pH-dependent manner; FcRn engagement at pH 5.0–6.0 in acidified endosomes results in sorting of the FcRn-IgG complex to the cell surface, where exposure to physiological pH of 7.4 triggers ligand dissociation [6, 7]. In this manner, FcRn rescues IgG from intracellular degradation and regulates the systemic pharmacokinetics and biodistribution of Fc-containing antibody biologics [8–11]. To avoid systemic exposure, the Fc fragment of faricimab is engineered to neutralize FcRn binding by introducing 3 amino acid substitutions: I253A, H310A, and H435A [3, 12]. Accordingly, we used ELISA to verify that faricimab maintained absence of human FcRn binding following withdrawal and storage in the syringe. Because bevacizumab contains a wild-type human IgG1 Fc, it was included as a positive control.

We coated 96-well EIA/RIA 3590 plates (Corning, Corning, NY) with 100-µl 0.5-µg/mL human VEGF165 (Sino Biological) and blocked them before adding 2000-ng/mL faricimab and bevacizumab from the stock solutions prepared for the VEGF-binding ELISA described above. Next, we washed the plates with buffer pH 5.5 (100-mM phosphate buffer, 0.15-M NaCl, 4% skimmed milk, 0.05% Tween 20) or S/PBS/T pH 7.4. All subsequent washing steps were carried out with said buffers of either pH 5.5 or 7.4. Following washing, 100-µl glutathione-S transferases (GST)-fused recombinant human FcRn was added at a final concentration of 1 µg/mL diluted in S/PBS/T with either pH 5.5 or pH 7.4. After another round of washing, horseradish peroxidase-conjugated anti-GST (Rockland Immunochemicals Inc) diluted 1:8000 in either pH 5.5 or pH 7.4 was added followed by incubation for 1 h at RT on a shaker. After a final wash, bound receptor was visualized by adding 100-µl tetramethylbenzidine substrate (Merck) followed by 100-µl 1-M HCl after 30 min. The absorbance at 450 nm was measured using a Sunrise spectrophotometer (Tecan Group Ltd.).

### Statistics

To address whether withdrawal of faricimab into the syringe affected the antibody, we compared vial samples to D0-n with the unpaired Student’s *t*-test. When addressing whether storage of faricimab in the syringe affected the antibody, D7-n, D7-c, D14-c, and D37-c were compared with D0-n with the Welch and Brown-Forsythe one-way ANOVA tests.

## Results

### Concentration measurements

**Fig. 2:**
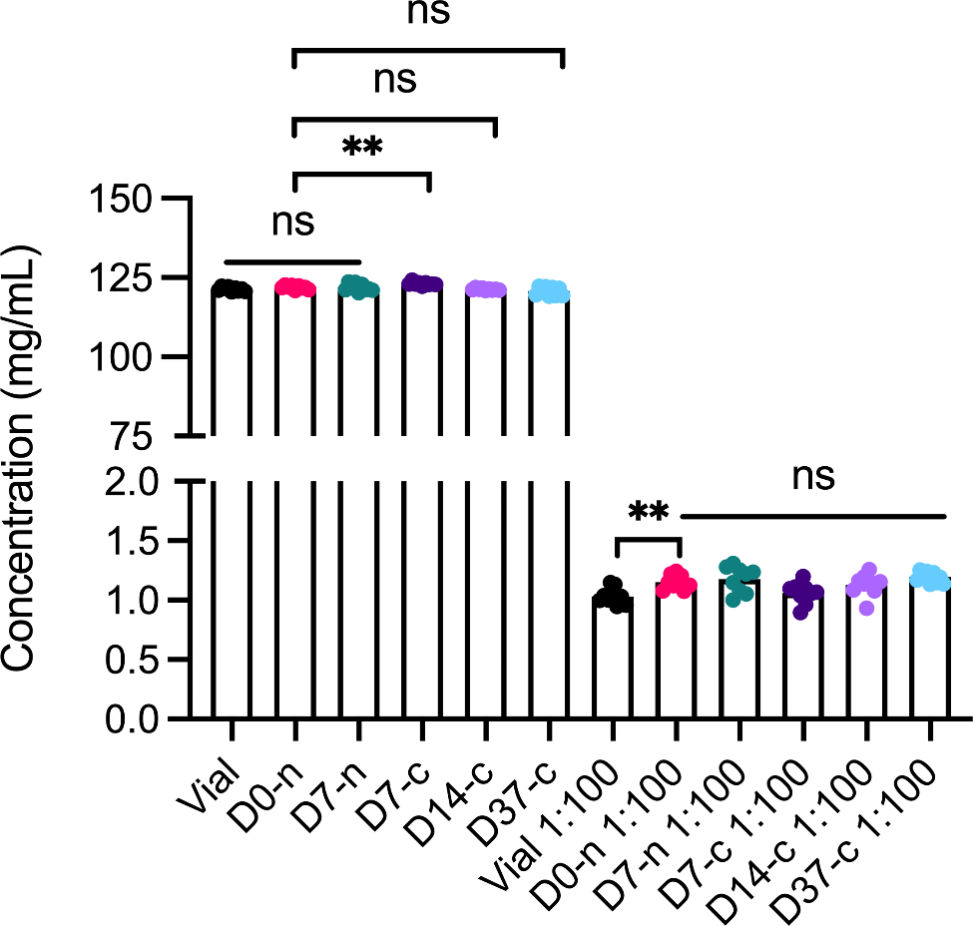
Concentrations of faricimab directly from the vial and after withdrawal and storage in the Zero Residual syringe. The figure shows both undiluted and diluted samples (diluted 1:100 in sterile PBS). n = 9 for all sample sets. **p = < 0.005, ns = non-significant

We used spectrophotometry to assess whether withdrawal and storage in the syringe affected faricimab sample concentration (Fig. 2). For non-diluted samples, D0-n (121.9 mg/mL) showed lower concentration than D7-c (123.1 mg/mL). For diluted samples, D0-n samples (1.15 mg/mL) showed higher concentration than vial samples (1.03 mg/mL). Taken together, these differences were minor and not consistent between undiluted and diluted samples.

### Protein stability

**Fig. 3:**
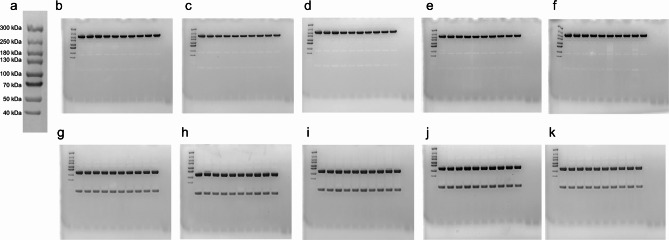
Faricimab ran on SDS-PAGE next to a **(a)** Spectra Multicolor High Range Protein Ladder under non-reducing **(b-f)** and reducing conditions **(g-k)**. For all runs, the ladder is found to the left, followed by a vial sample and either 9 D0-n samples **(b, g)**, D7-n samples **(c, h)**, D7-c samples **(d, i)**, D14-c samples **(e, j)**, or D37-c samples **(f, k)**. As a reference, we ran the gels next to a Spectra Multicolor Broad Range Protein Ladder after the study day, to confirm the size with a lower range standard (Supplementary material). D = day, n = needle, c = cap

We assessed the sample integrity of faricimab following withdrawal and storage in the syringe by SDS-PAGE under non-reducing and reducing conditions (Fig. 3). The results did not reveal differences in protein migration and band intensity between the different time points when applying samples on non-reduced gels (Fig. 3b-f), as the proteins migrated in accordance with their expected molecular mass of approximately 150 kDa. When the samples were run under reducing conditions (Fig. 3g-k), the proteins migrated as main bands corresponding to approximately 50 kDa for the heavy chains and 25 kDa for the light chains, as expected.

To confirm the size of the light chain, we repeated all gels and ran them 5 days after the study day, next to a Spectra Multicolor Broad Range Protein Ladder (**Supplementary material**). This confirmed the size of all bands as seen on the study day. In short, faricimab migrated in line with the expected molecular mass under both reducing and non-reducing conditions, independent of whether it originated directly from a vial, from a syringe-needle withdrawn on the day of the study or stored for 7 days, or from a syringe-cap stored for 7, 14, or 37 days. We did not detect degradation products or aggregation. The raw images can be found in **Supplementary material.**

**Fig. 4:**
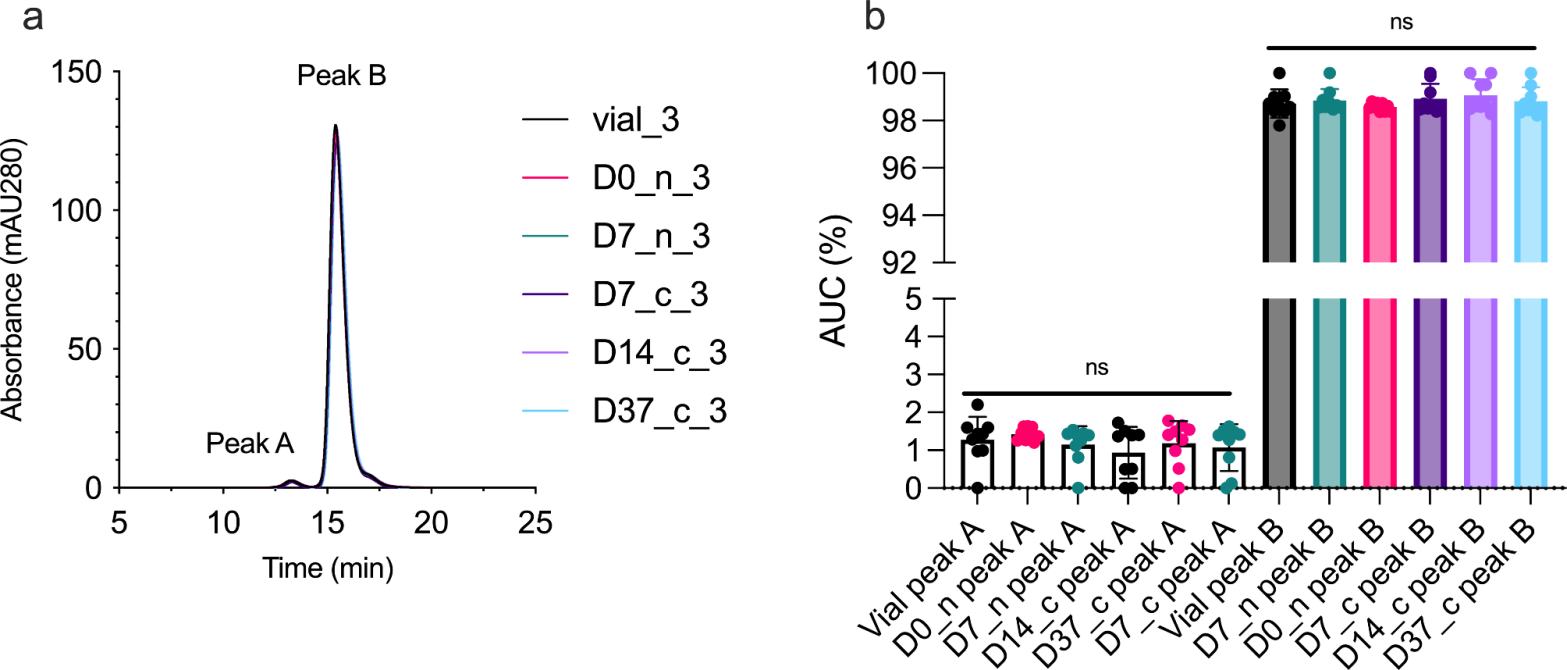
SEC of faricimab. **(a)** Representative chromatograms for vial, D0-n, D7-n and -c, D14-c, and D37-c are displayed as start and endpoint storage conditions. **(b)** We calculated the area under curve for all 9 syringes for vial, D0-n, D7-n and -c, D14-c, and D37-c. ns = non-significant, D = day, n = needle, c = cap

Next, we measured the propensity of faricimab to aggregate in a non-covalent manner following withdrawal and storage in the syringe by SEC (Fig. 4). We found that all samples of faricimab had a major elution peak preceded by a smaller, additional peak, displaying an elution profile similar to that observed for bevacizumab under the same conditions [2]. All elution histograms were practically identical, with only minor differences in absorbance. Vial-3, D0-n_3, D7-n_3, D7-c_3, D14-c_3, and D37-c_3 are shown as a representative experiment (Fig. 4a). Statistical comparison of the area under curve of the individual peaks did not demonstrate significant differences (Fig. 4b).

**Fig. 5:**
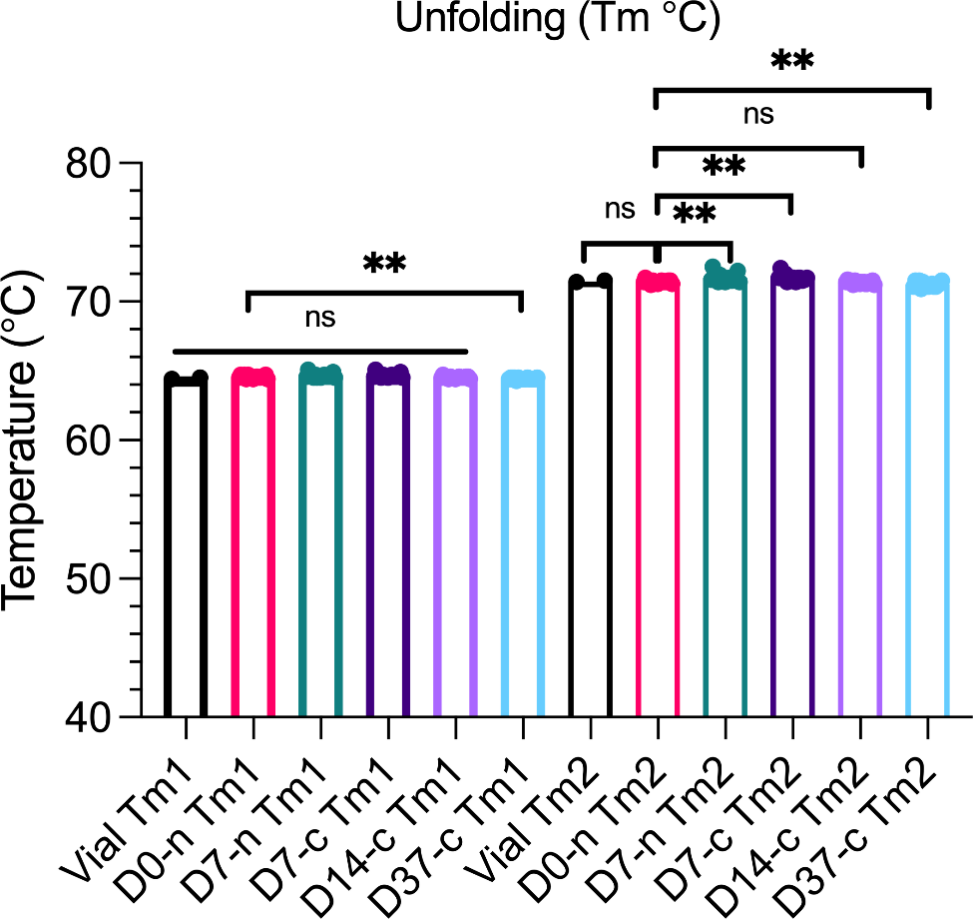
Melting temperatures (Tm) of faricimab. **=p < 0.005, ns = non-significant

As a final measure of protein stability, we performed nano DSF to determine the thermal stability of faricimab (Fig. 5). The melting process of faricimab occurred in two steps because of its heavy and light chains, with respective Tms of approximately 64 and 71.5 °C. No significant differences between D0 and vial samples at either Tm1 or Tm2 were measured. However, we did observe significant variations when comparing syringes stored for different times (D7-n, D7-c, and D37c) to D0-n, but these differences were very minor. Comparison of D14-c and D0-n did not reveal significant differences. Notably, no additional events were observed when increasing the temperature to 95 °C. We excluded data points from statistical analysis if they differed from the sample group mean by > 10 °C.

### Antigen binding properties

**Fig. 6:**
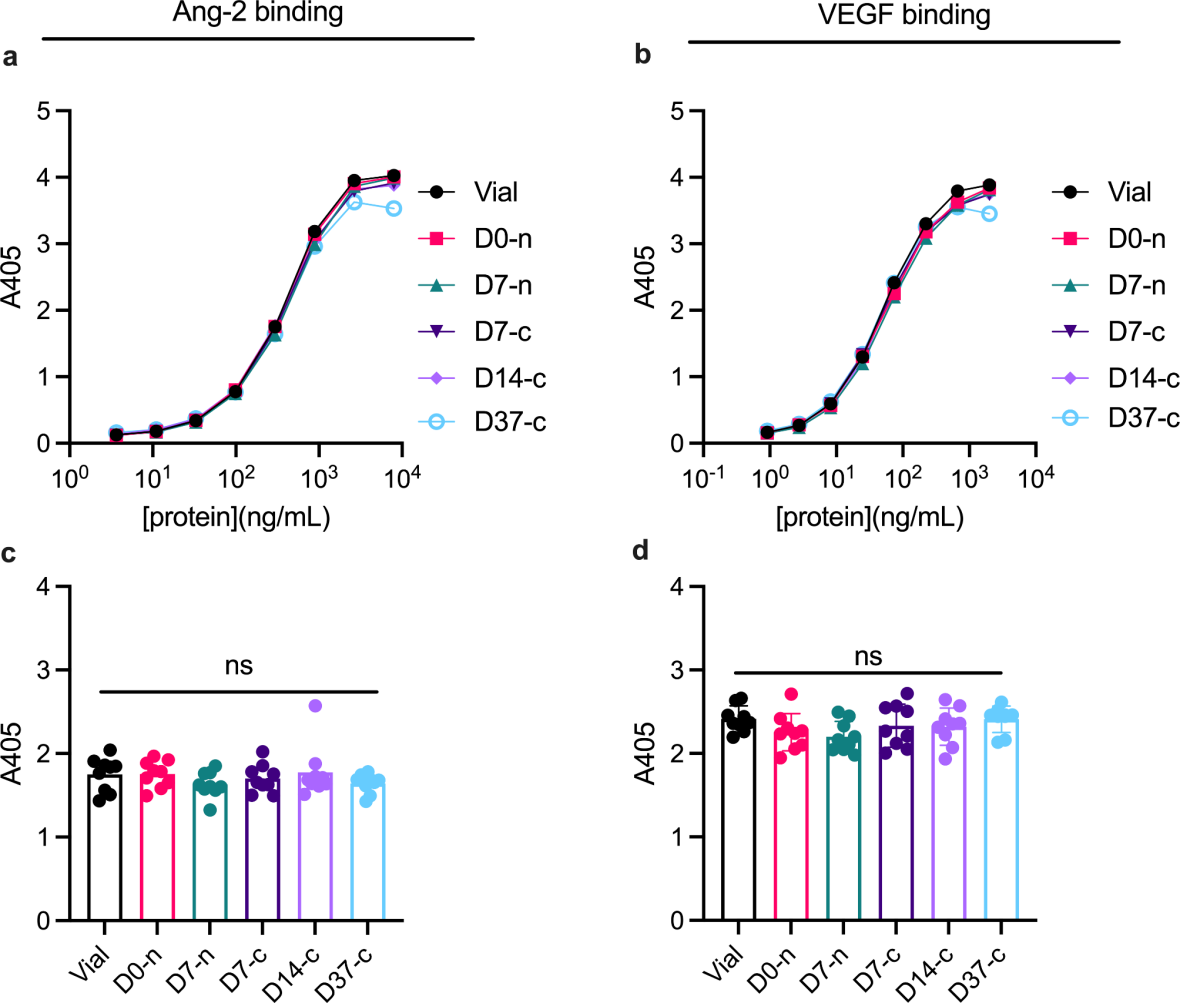
Antigen binding properties determined by ELISA. **(a-b)** Binding of titrated amounts of faricimab to **(a)** Ang-2 and **(b)** VEGF. The binding curves in **(a-b)** represent the average of individual binding curves from each sample group (averaged from n = 9 per curve). We retrieved single values from the exponential phase of the binding curves for statistical analyses, at **(c)** [faricimab] = 296.04 ng/mL for Ang-2 and **(d)** [faricimab] = 74.04 ng/mL for VEGF. ns = non-significant

We used ELISA to assess whether syringe withdrawal and storage in the syringe affected binding of faricimab to its cognate antigens: Ang-2 and VEGF-A (Fig. 6). Capture of titrated amounts of faricimab on either Ang-2 or VEGF-A was detected by means of an anti-human Fc antibody and yielded overlapping binding curves (Fig. 6a and b). Statistical analysis did not reveal significant differences in Ang-2 (Fig. 6c) or VEGF-A binding (Fig. 6d) between timepoints.

### FcRn binding properties

**Fig. 7:**
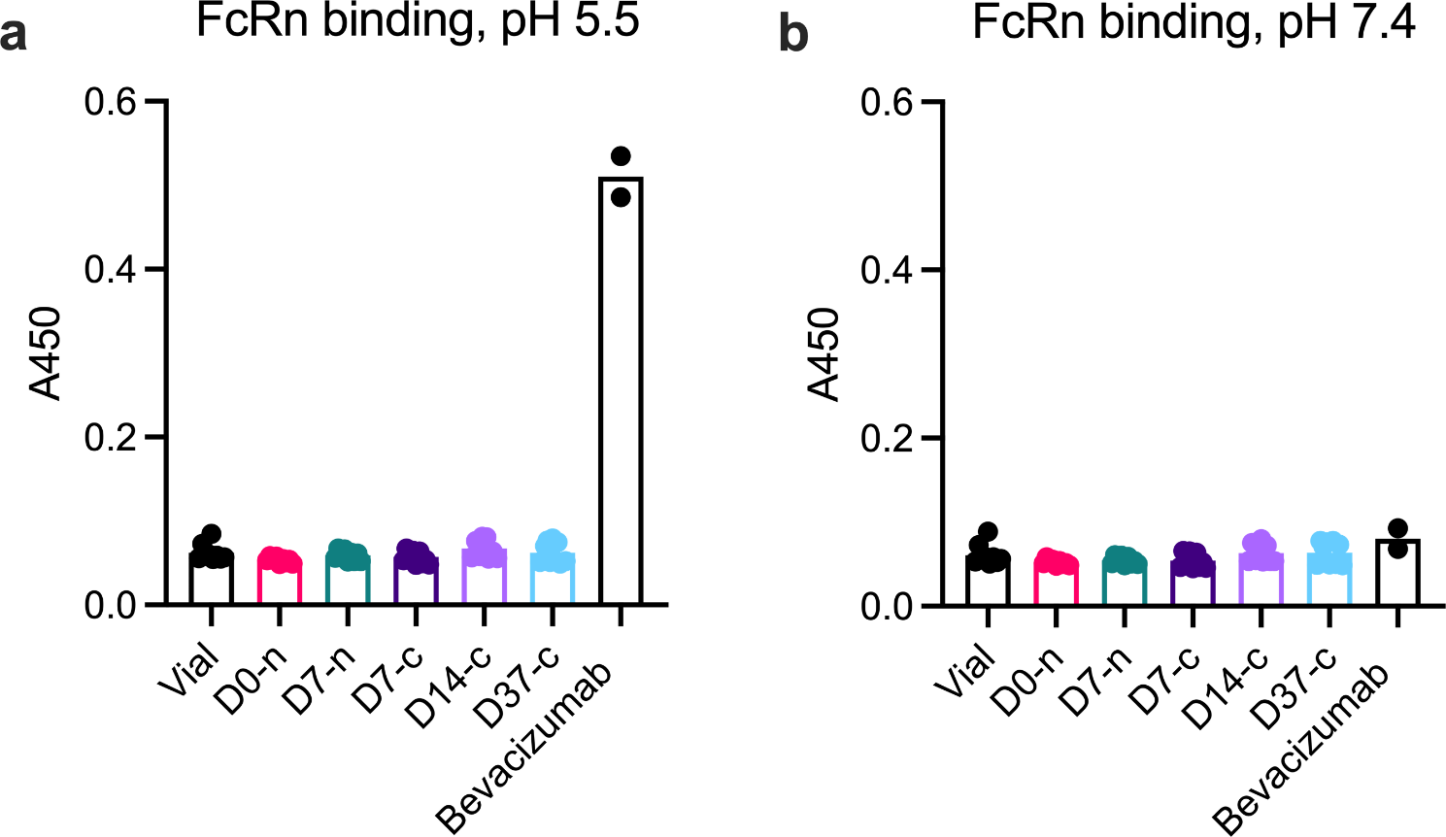
FcRn binding properties determined by ELISA. **(a)** No binding of faricimab to human FcRn at pH 5.5. Bevacizumab functions as positive control. **(b)** No binding of either faricimab or bevacizumab at pH 7.4. Each data point represents the mean of a duplicate point on the ELISA plate

We used ELISA to verify that faricimab did not bind FcRn following withdrawal and storage in the syringe, and used bevacizumab, which has an intact FcRn binding site, as a positive control (Fig. 7). As expected, faricimab did not bind FcRn (Fig. 7a), whereas bevacizumab bound FcRn at acidic pH but not at pH 7.4 (Fig. 7b).

## Discussion

Pharmaceutical compounding and storage of prefilled syringes for IVI is common practice in ophthalmology but may disturb the properties of antibody biologics. Faricimab is a new therapeutic antibody that not only binds VEGF-A but also Ang-2. This study investigated the effect of withdrawal and storage of faricimab in the Zero Residual silicone oil-free, 0.2-mL syringe. We found that the procedure did not negatively affect concentration, integrity, aggregation, or thermal stability of faricimab. Importantly, faricimab maintained its VEGF-A and Ang-2 binding properties through 37 days and did not bind FcRn under any conditions. We conclude that pharmaceutical compounding and storage of faricimab in syringes do not impair the structure or function of the bi-specific therapeutic antibody.

Pharmaceutical compounding of prefilled syringes for IVI can optimize hygiene standards, save clinician time, and allow for splitting of vials to reduce costs. At the same time, it is important to realize that the practice introduces a possibility for drug-syringe interactions, highlighting the importance of selecting an appropriate syringe. The Zero Residual syringe incorporates several favourable features: high accuracy and precision, no silicone oil, Luer Lock (i.e., no fixed needle), and negligible dead volume [1]. A previous study from our group also showed that it could store bevacizumab, ranibizumab, and aflibercept without compromising their functional binding, stability or FcRn-mediated cellular recycling [2]. However, because faricimab differs from these antibody biologics by being a first-in-class bispecific antibody against not only VEGF-A but also Ang-2, the results supporting pharmaceutical compounding of other biologics cannot be extrapolated to faricimab. Instead, it is necessary to study drug-syringe interactions for faricimab specifically, and to include methods for measuring the effect of pharmaceutical compounding and storage on both VEGF-A and Ang-2 binding properties.

An important concern about compounding and storage of biologics is the possibility of protein aggregation and sample loss, which could potentially reduce the clinical effect and cause sterile inflammation [13]. We used several methods to assess the integrity, stability, and aggregation of faricimab: concentration measurements, SDS-PAGE, SEC, and DSF. SDS-PAGE and SEC did not reveal impurities. The thermal stability and concentration of faricimab varied slightly for different storage times, but the differences were minor (within the range of ±0.3 ^°^C and 1.2 mg/mL) and not consistently related to storage time or sample groups. Taken together, we believe the slight variation in Tm and concentration we observed was merely due to methodological variation, and we conclude that compounding and storage did not significantly affect the stability of faricimab.

Unquestionably, the most central aspect of antibody biologics is their ability to bind their targets. In this regard, faricimab has a novel mechanism of action as it binds Ang-2 in addition to VEGF-A via two distinct Fab arms coupled to one IgG1-Fc fragment. To verify consistent monovalent binding of faricimab to VEGF-A, we used a VEGF-A-specific ELISA, as previously reported [11, 14]. To study Ang-2 binding, we also established an ELISA where faricimab could be captured on recombinant human Ang-2 coated in wells. The combination of these two ELISA methods robustly visualized that compounding and storage of faricimab for up to 37 days did not affect its bispecific antigen-binding properties. As the two ELISA methods involved detection of bound faricimab by use of an anti-Fc antibody, they also confirmed structural integrity of the IgG1 Fc fragment.

We have previously shown that syringe withdrawal and storage may affect FcRn binding of the IgG1-Fc-containing therapeutics aflibercept and bevacizumab, but this finding did not translate into differences in FcRn-mediated cellular recycling [2]. Faricimab, on the other hand, has a modified Fc fragment that neutralizes FcRn binding, enabling a short systemic half-life [3, 15]. We addressed whether syringe withdrawal and storage could diminish this quality by causing unspecific binding to FcRn. We found that faricimab did not bind FcRn, i.e., the compounding procedure did not alter the modified Fc fragment of faricimab.

Some study limitations should be noted. First, as this was an in vitro study, we did not test the prefilled syringes clinically. Second, while we prefilled the syringes under controlled aseptic conditions, we did not evaluate the sterility of the prefilled syringes. Still, we would like to point out that a pharmaceutical compounding procedure must not only ensure drug stability, which was the focus of this study, but also sterility and microbiological safety.

In conclusion, withdrawal and storage of faricimab in syringes for up to 37 days did not impair the stability and bi-specific binding properties of the therapeutic antibody.

### Electronic supplementary material

Below is the link to the electronic supplementary material.

Supplementary Material 1

## Data Availability

The dataset is available upon reasonable request.

## Abbreviations

IVI: Intravitreal injection
VEGF-A: Vascular Endothelial Growth Factor A
Ang-2: Angiopoietin-2
SEC: Size-Exclusion Chromatography
Nano DSF: Nano Differential Scanning Fluorimetry
Tm: Melting Temperature
ELISA: Enzyme-Linked Immunosorbent Assay
RT: Room Temperature
ALP: Alkaline Phosphatase
GST: Glutathione-S Transferase
FcRn: Neonatal Fc Receptor
Fab: Fragment Antigen Binding
