# Supplementary material for: Pharmaceutical compounding and storage of faricimab in a syringe for intravitreal injection do not impair stability and bi-specific binding properties
Source: Int J Retina Vitreous. 2023 Nov 7;9:65. doi: 10.1186/s40942-023-00507-3 (PMC10631190; doi:10.1186/s40942-023-00507-3)

Supplementary material

The following figures show all obtained raw images of the SDS-PAGE gels. For all gels, the ladder is found to the left, followed by a vial sample. Note, we used a Spectra Multicolor High Range Protein Ladder on the day of experiment. To confirm the size of the light chain, we repeated all gels and ran them 5 days after the study day.

The samples were divided in the following way between gels:

# Gel,<br>non reduced	Samples	# Gel,<br>reduced	Samples
1	Day 0 from syringe	1 R	Day 0 from syringe
2	Day 7 from syringe	2 R	Day 7 from syringe
3	Day 7 from cap	3 R	Day 7 from cap
4	Day 14 from cap	4 R	Day 14 from cap
5	Day 37 from cap	5 R	Day 37 from cap

Study-day gels (using Spectra Multicolor High Range Protein Ladder)

Gel 1:

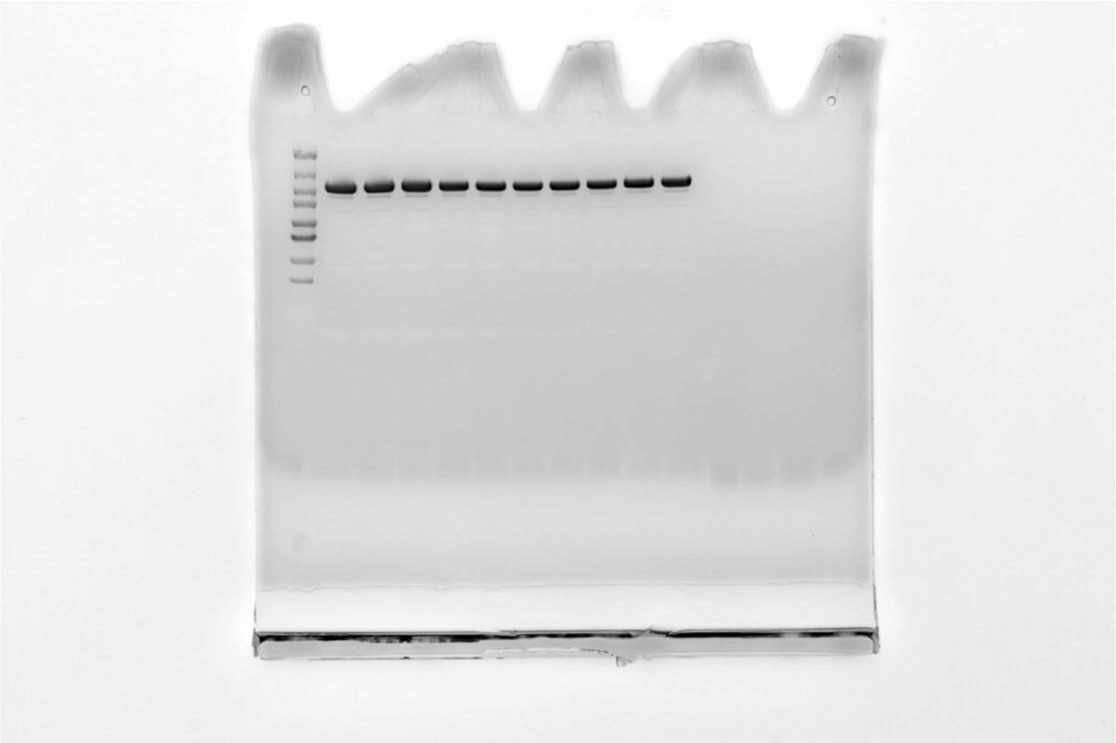

Gel 2:

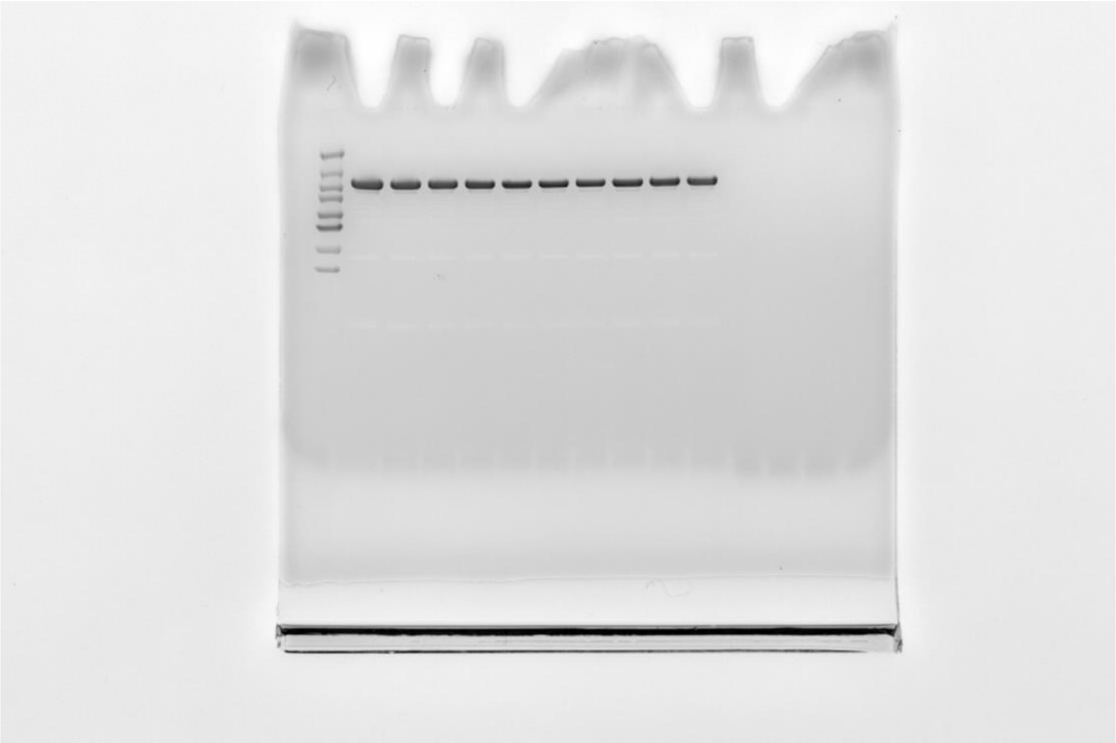

Gel 3:

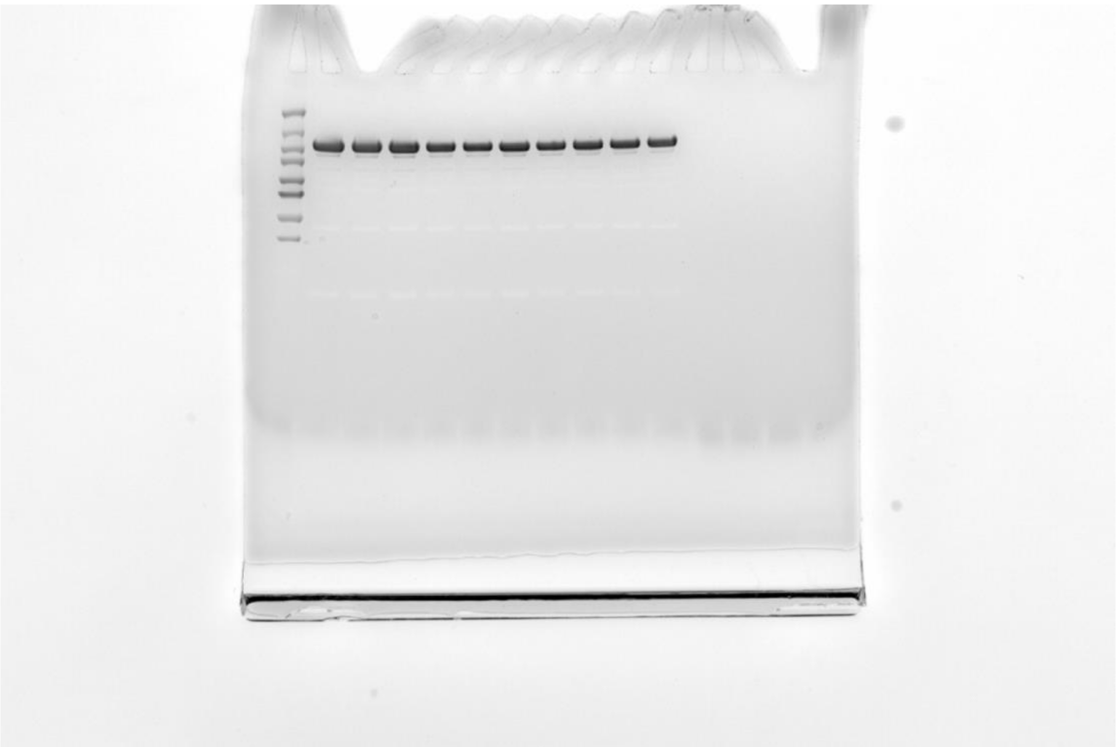

Gel 4:

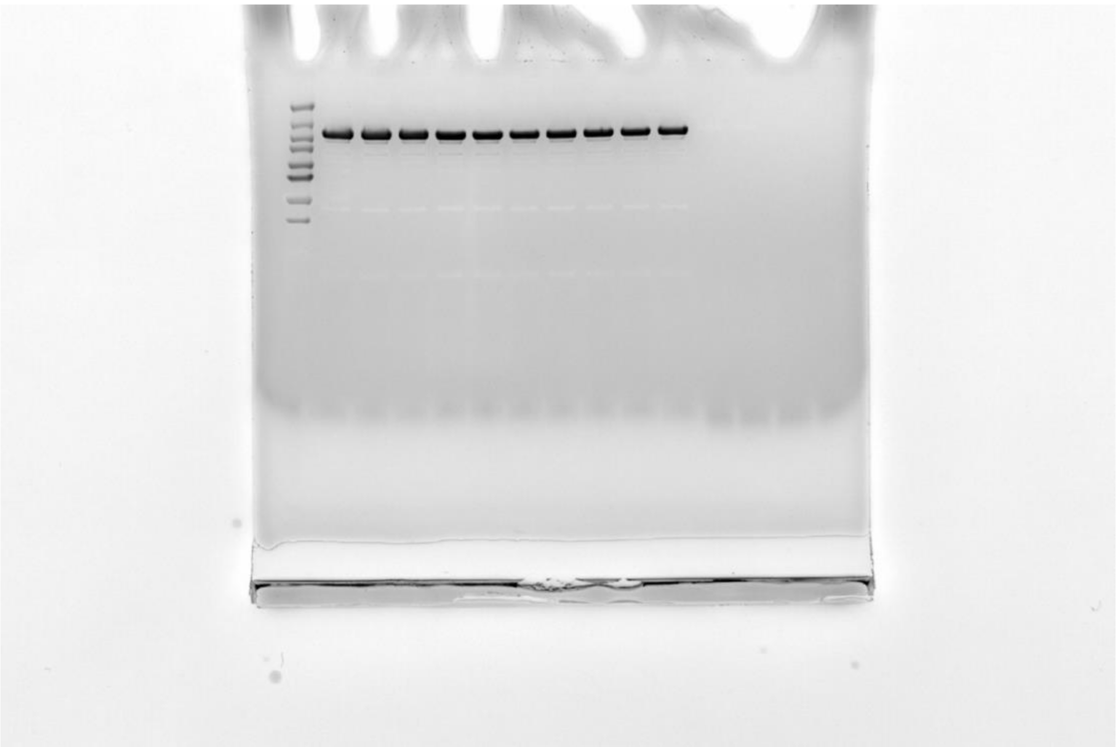

Gel 5:

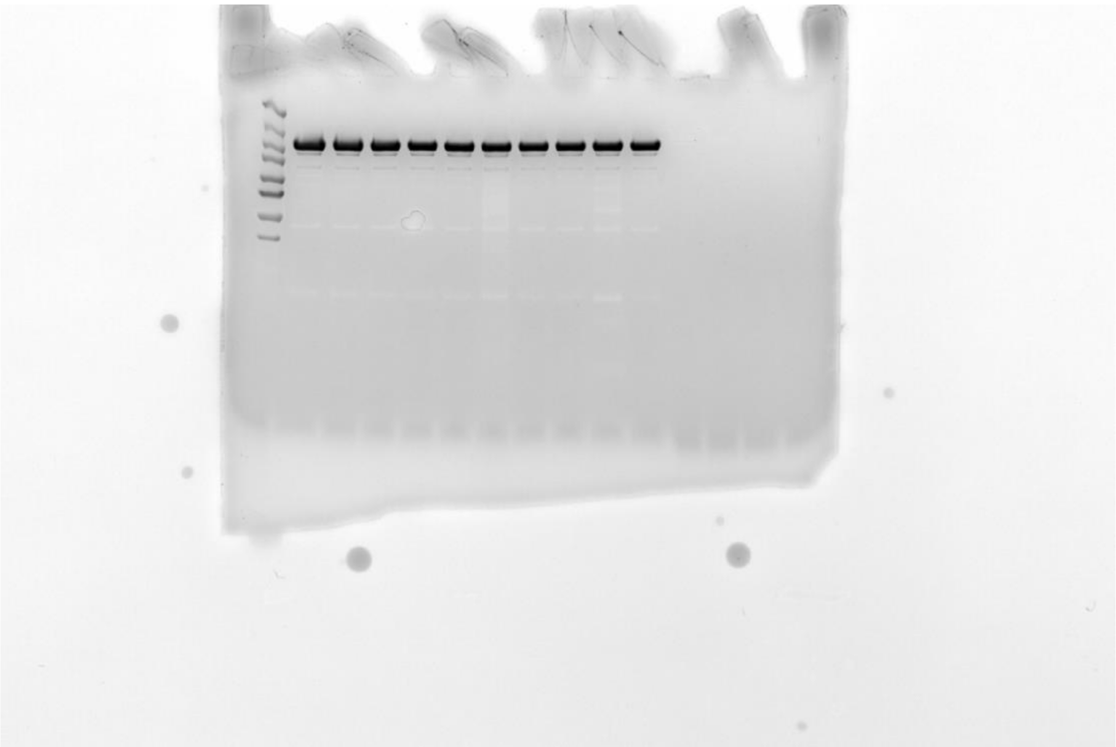

Gel 1 R:

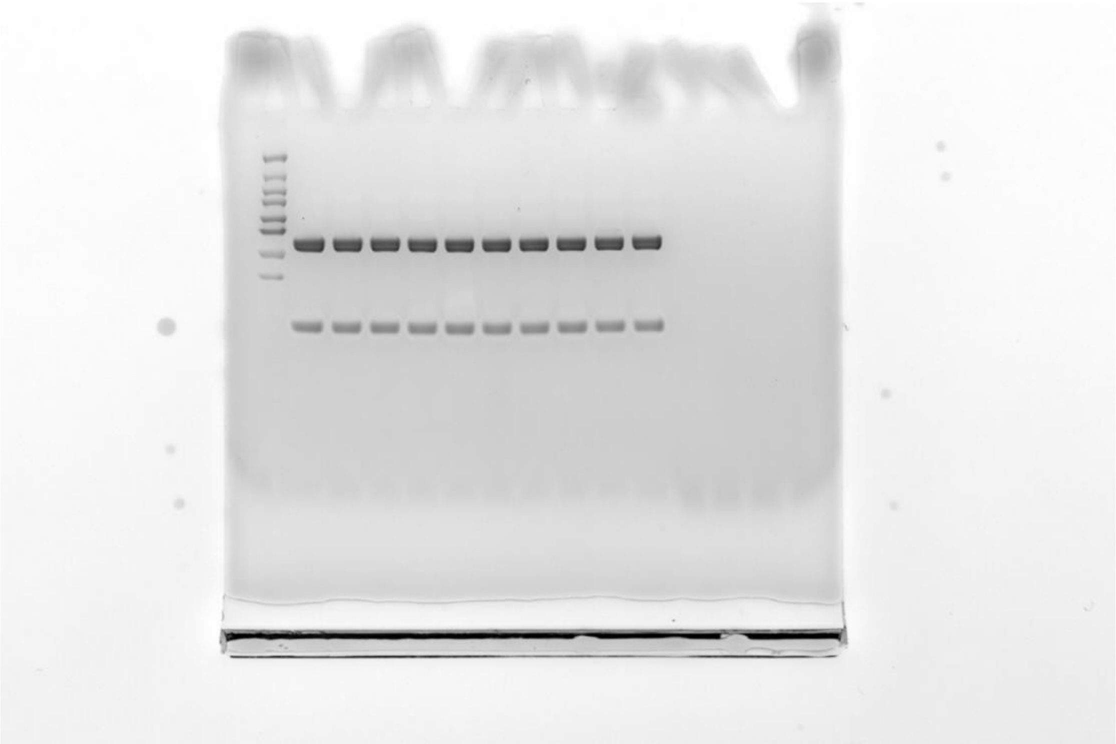

Gel 2 R:

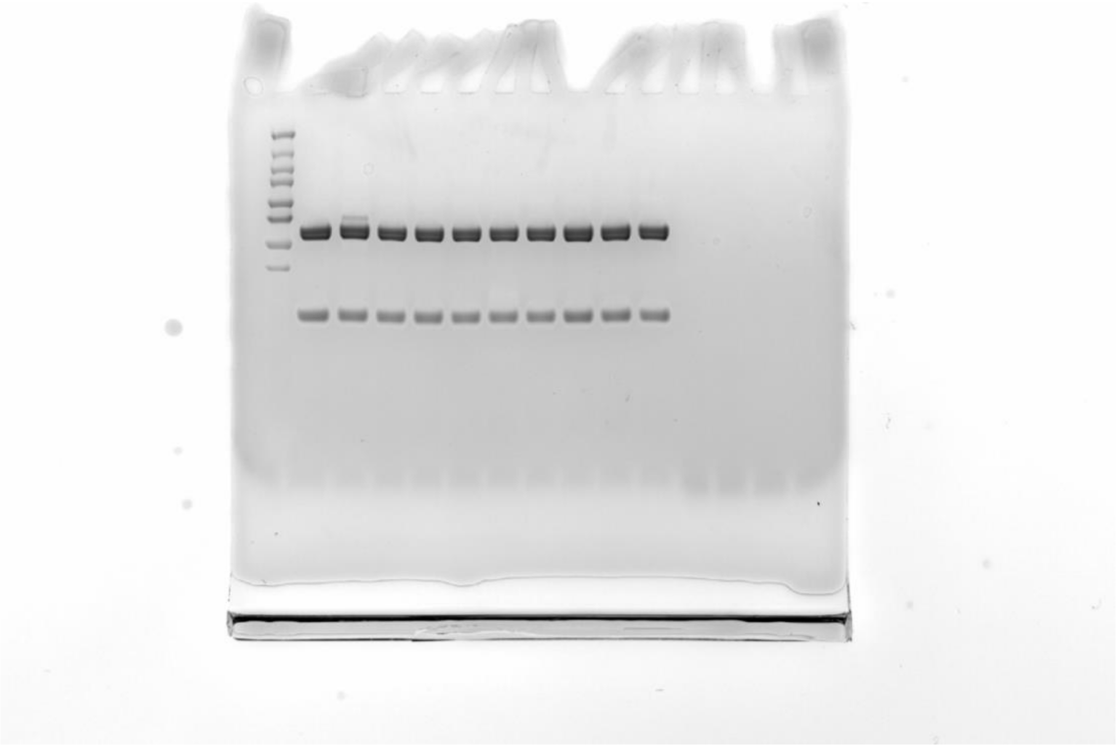

Gel 3 R:

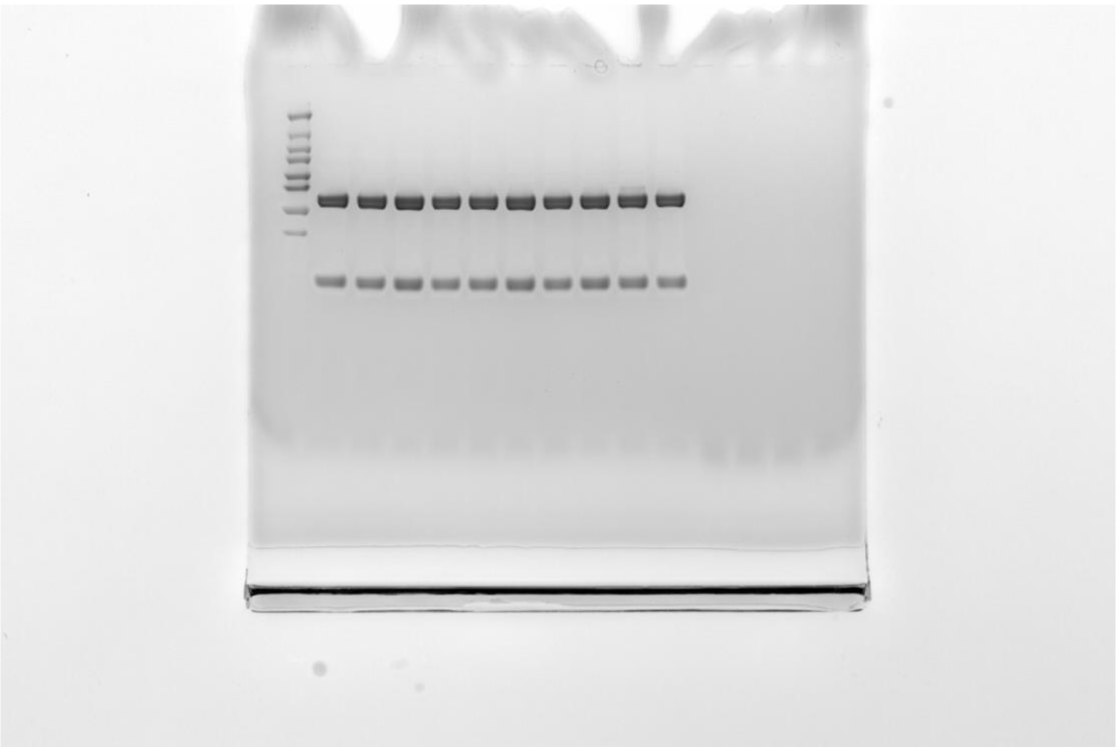

Gel 4 R:

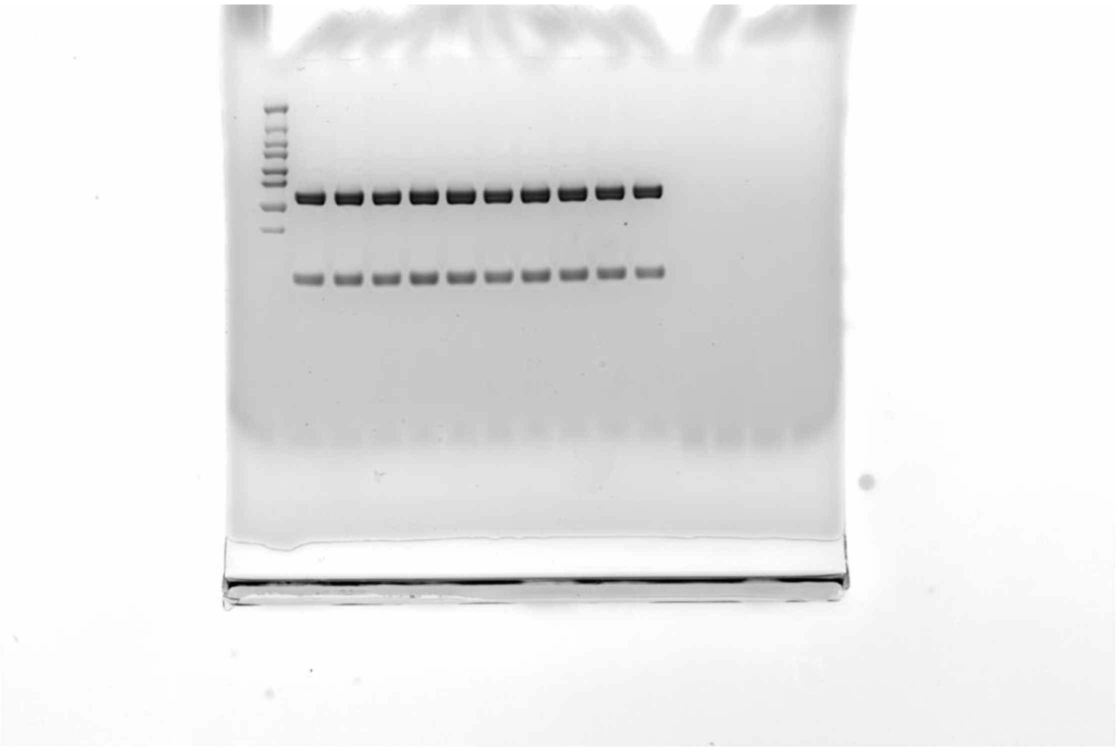

Gel 5 R:

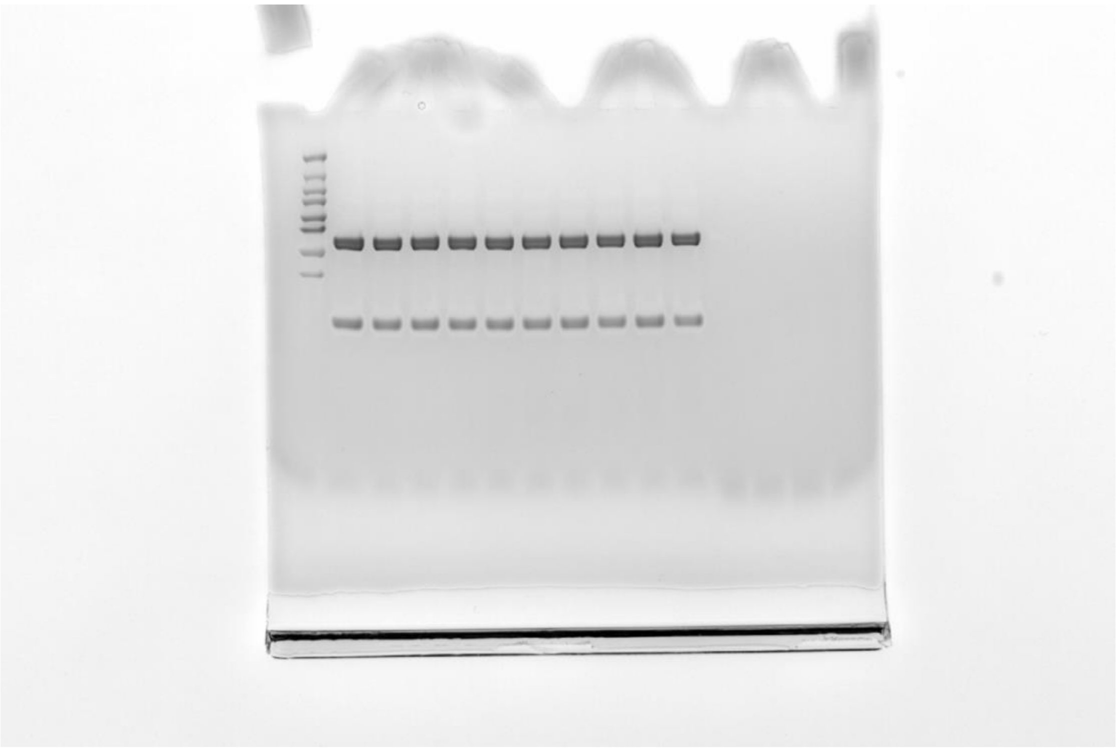

Day-5 gels (using Spectra Multicolor Broad Range Protein Ladder)

Gel 1:

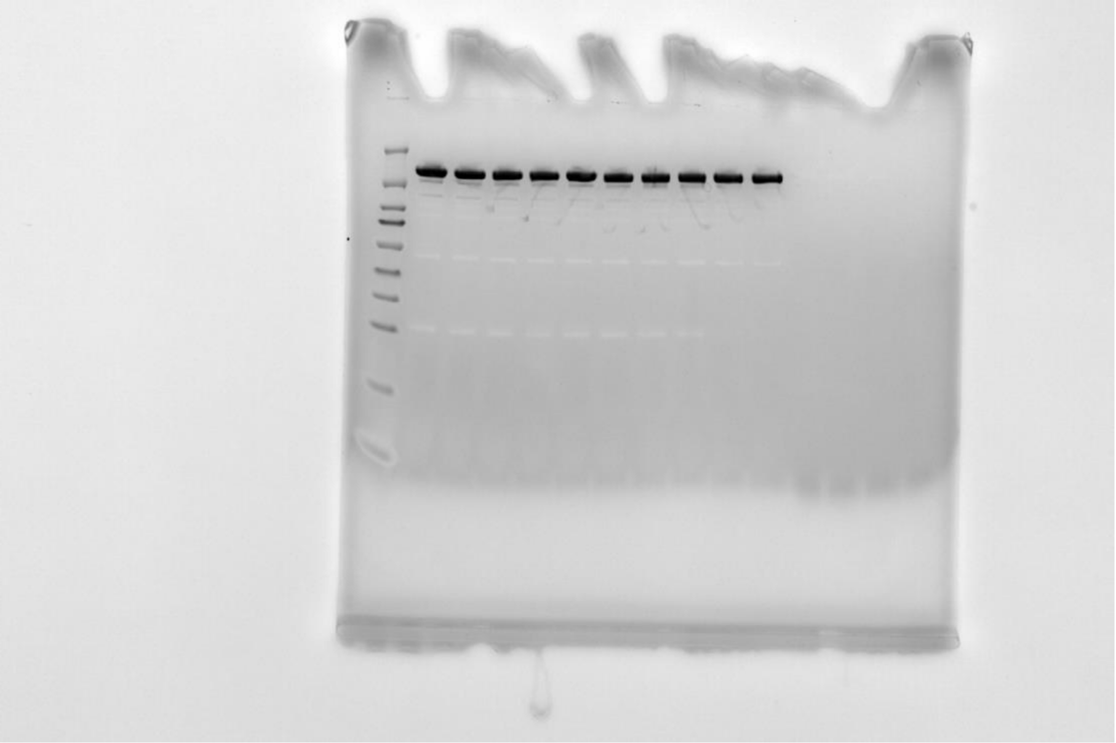

Gel 2:

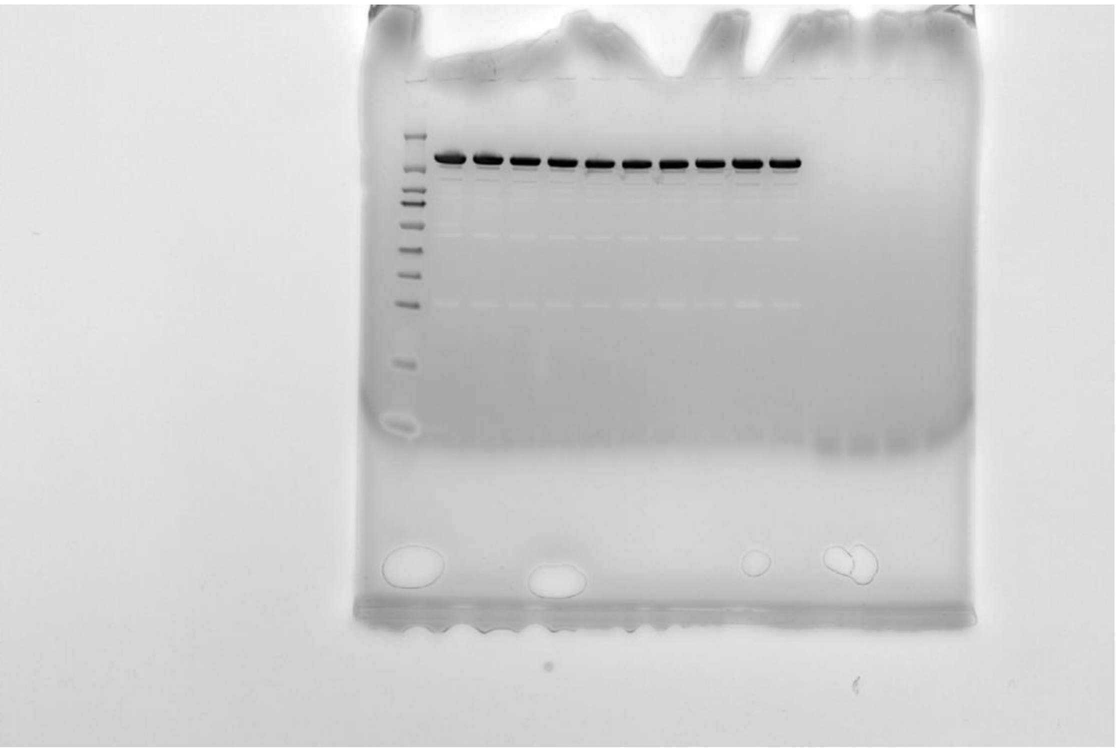

Gel 3:

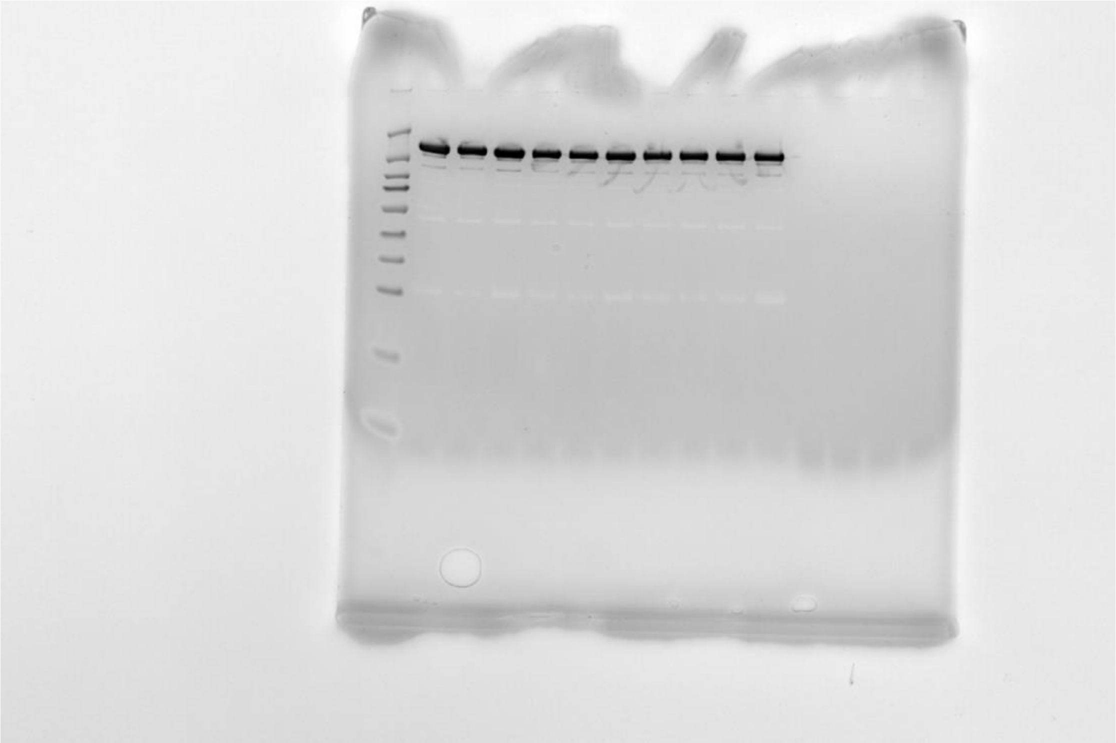

Gel 4:

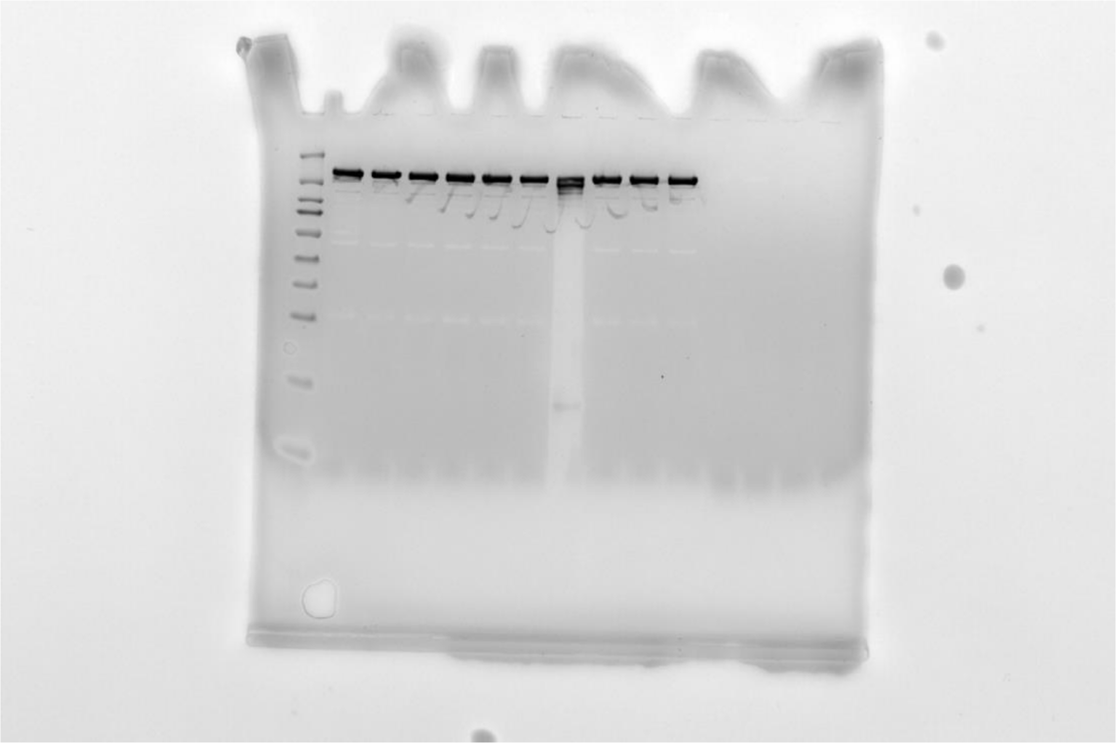

Gel 5:

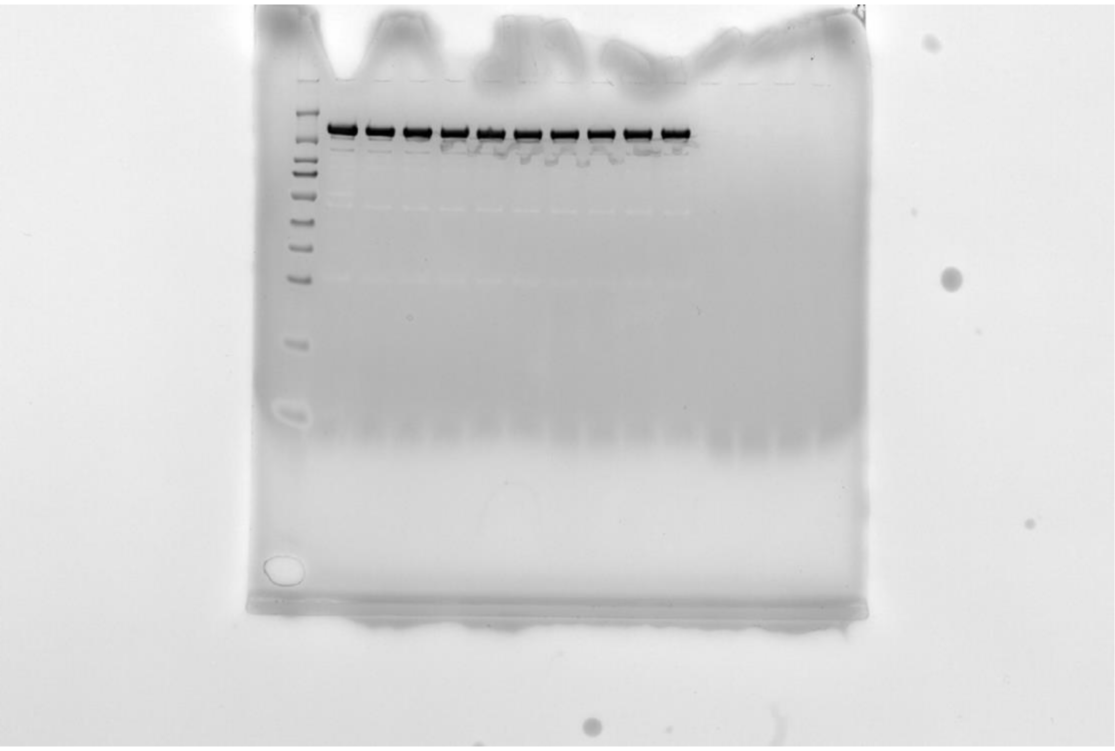

Gel 1 R:

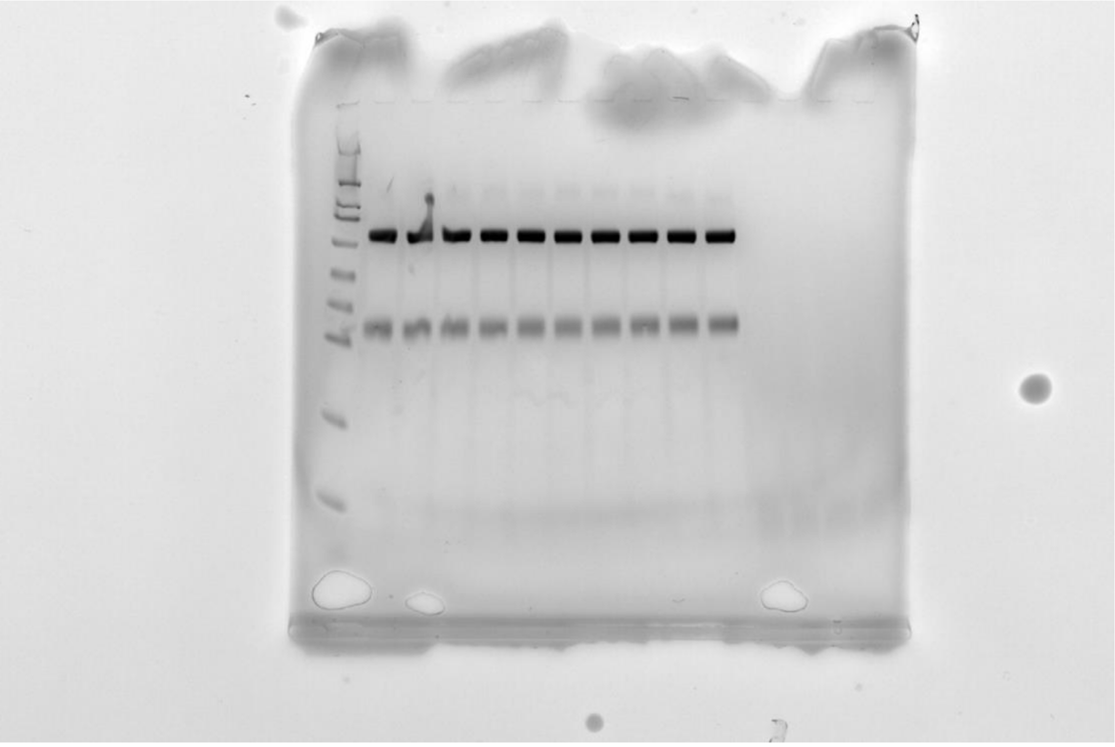

Gel 2 R:

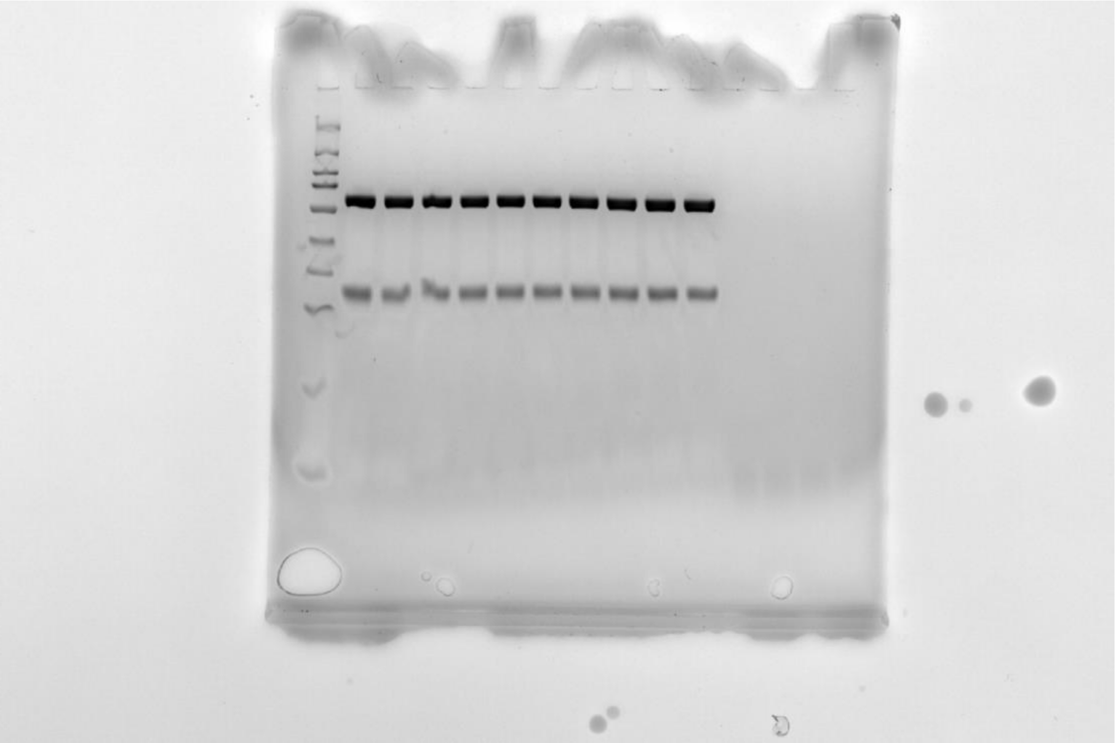

Gel 3 R:

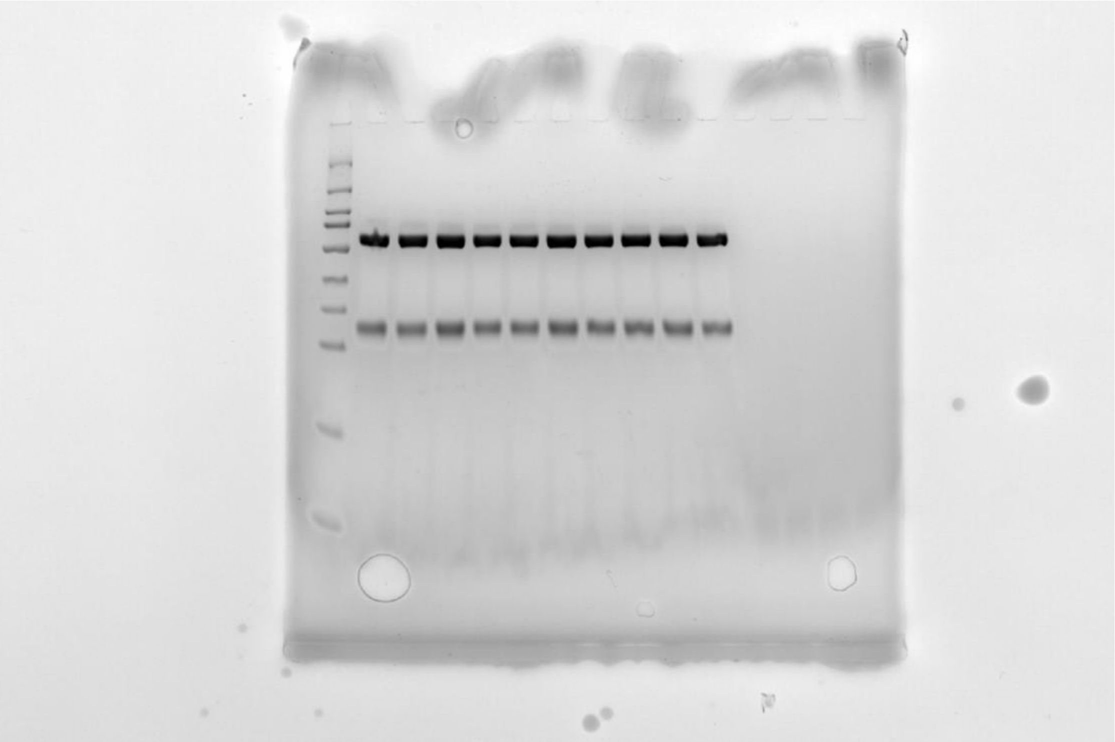

Gel 4 R:

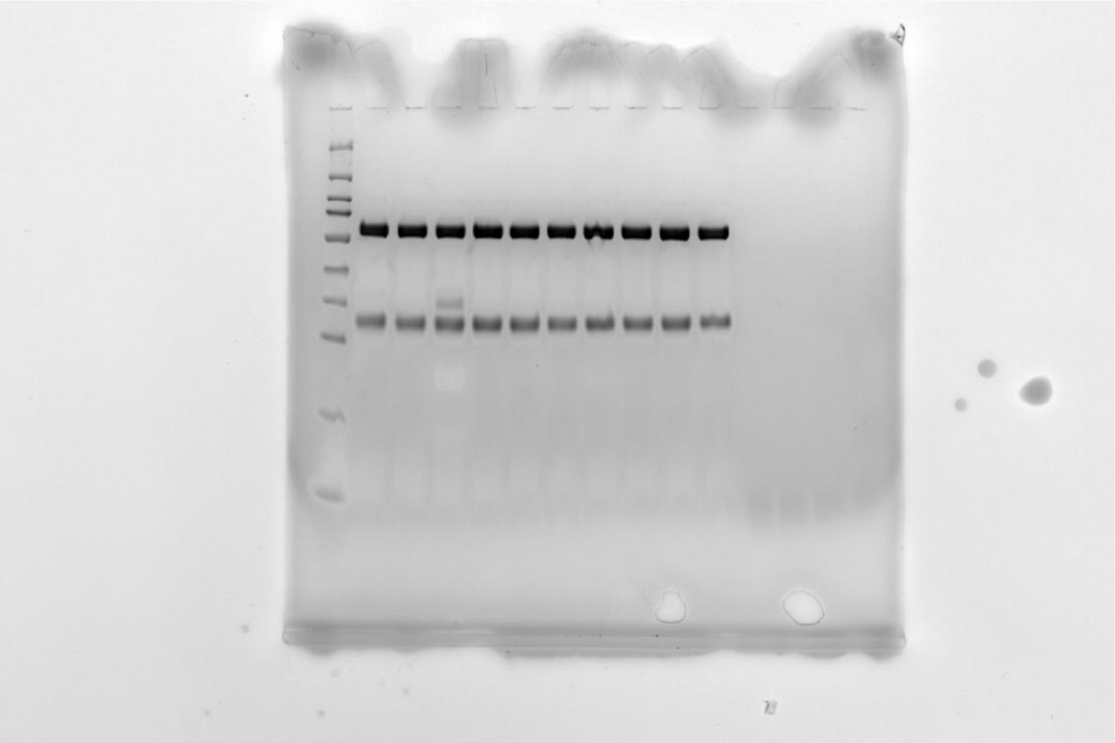

Gel 5 R:

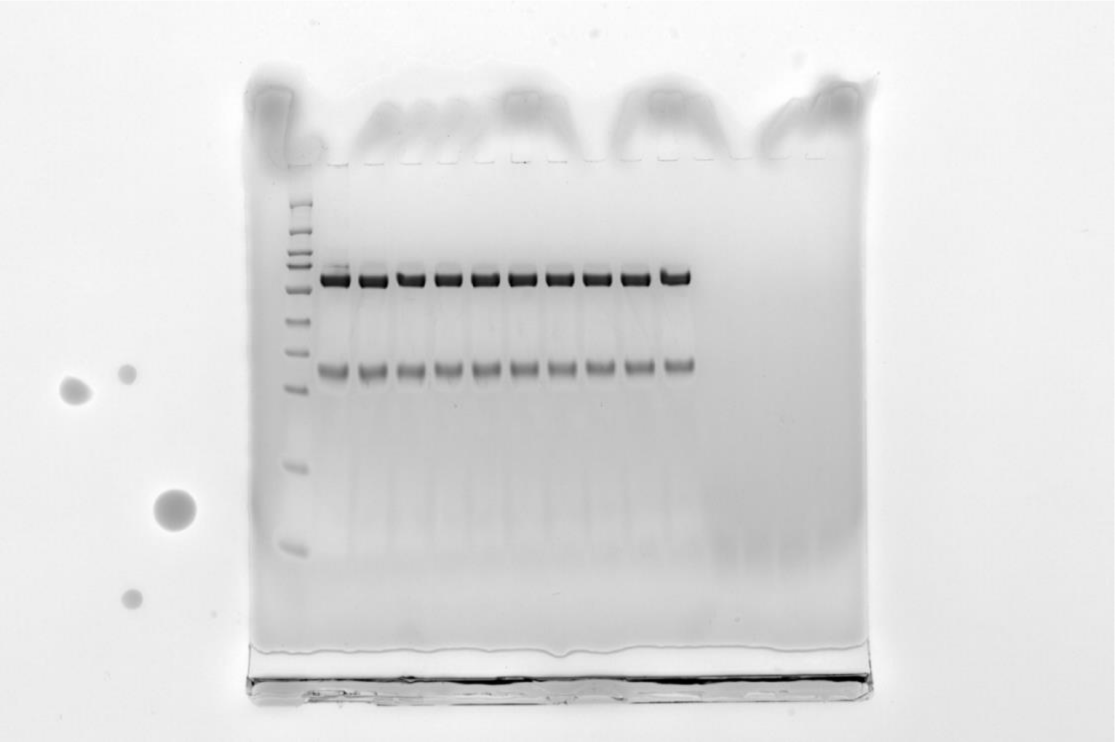

Supplement: Supplementary file 1 — Supplementary Material 1 [file 40942_2023_507_MOESM1_ESM.pdf]
